# Macrophage metabolic reprogramming and atherosclerotic plaque microenvironment: Fostering each other?

**DOI:** 10.1002/ctm2.1257

**Published:** 2023-05-07

**Authors:** Qi Xiao, Rongyao Hou, Linlin Xie, Mengying Niu, Xudong Pan, Xiaoyan Zhu

**Affiliations:** ^1^ Department of Neurology The Affiliated Hospital of Qingdao University Qingdao China; ^2^ Department of Neurology The Affiliated Hiser Hospital of Qingdao University Qingdao China; ^3^ Department of Critical Care Medicine The Affiliated Hospital of Qingdao University Qingdao China

**Keywords:** atherosclerosis, macrophage, metabolic reprogramming, plaque microenvironment

## Abstract

Macrophages are the central immune cells in atherosclerosis (AS) and play a critical role in the initiation, progression and invasion of atherosclerotic plaques. Metabolic reprogramming is a crucial feature that determines macrophage function and is driven by a combination of intrinsic alterations in macrophages and extrinsic factors such as cytokines acting in the plaque microenvironment. Intrinsic macrophage mechanisms activate signal transduction pathways that change metabolic enzyme activity, and the expression of metabolic regulators. Extrinsic signalling mechanisms involve lipids and cytokines in the microenvironment, promoting and amplifying macrophage metabolic reprogramming. This review describes the intrinsic and extrinsic mechanisms driving macrophage metabolic reprogramming in the AS microenvironment and the interplay of these metabolic rewires in the microenvironment. Moreover, we discuss whether targeting these different pathways to treat macrophage microenvironmental changes can alter the fate of the vulnerable plaques.

## Graphical abstract

1. Intrinsic and extrinsic mechanisms drive the metabolic reprogramming of macrophages in the AS microenvironment.

2. Macrophage metabolic reprogramming also interacts with the atherosclerotic plaque microenvironment.

3. Targeting macrophages and plaque microenvironment is expected to be potential diagnostic and therapeutic targets for plaques.

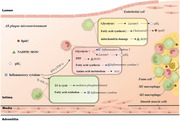

## 1 INTRODUCTION

Atherosclerosis (AS) is the formation of fibrofatty lesions in the intima of arteries.^1^ The word AS comes from the Greek word for ‘porridge’ or ‘gruel’. It graphically represents the appearance of the lipid core of a typical atherosclerotic plaque.^2^ Over time, atherosclerotic plaques become more fibrotic and accumulate calcium.^2^ Atherosclerotic plaques can trigger thrombosis via plaque rupture or plaque instability due to surface erosion or plaque encroachment into the arterial lumen, blocking blood flow and causing tissue ischaemia.^3^ The former is usually more acute, leading to high morbidity and mortality rates, including myocardial infarction and stroke.^4^ In contrast, the AS microenvironment overlying the fibrous cap is closely associated with plaque stability. Many cell types, including endothelial cells (ECs), macrophages and vascular smooth muscle cells (VSMCs), are involved in forming or are incorporated into the atherosclerotic plaque microenvironment.^5^, ^6^ However, the antecedent life of macrophages in microenvironmental changes is critical for atherosclerotic plaque stability. Therefore, understanding the dynamics of macrophages involved in AS onset, progression and regression is essential for slowing down plaque progression and influencing plaque stability.

Macrophages maintain tissue homeostasis, act as immune sentinels, and are the central immune cells in AS.^7^ Macrophages can reside in tissues independent of the haematopoietic system and can be maintained by self‐renewal in localised areas. Alternatively, they can originate from the monocyte system, which infiltrates tissues and differentiates in response to their microenvironment with remarkable plasticity and heterogeneity.^8^, ^9^ Macrophages involved in AS belong to the latter category, and monocytes recruited to arteries respond functionally to microenvironmental stimuli and signals, changing from a quiescent to an activated state.^10^ Emerging evidence has demonstrated that macrophage activation states consist of a continuum of phenotypes to meet the functional requirements, such as phagocytosis, proliferation and cytokine production.^11^ The two main macrophage subpopulations performing different functions characterise the extremes of the continuum activation state, including classically activated or inflammatory (M1) and alternatively activated or anti‐inflammatory (M2) macrophages.^11^, ^12^, ^13^ Macrophages phagocytose modified lipoproteins in the plaque microenvironment, converting them into cholesterol‐laden foam cells. Although macrophage clearance of lipoproteins may be beneficial at the beginning of this immune response, negative feedback is lacking after uptake; thus, these cells become heavily engorged with lipids.^5^, ^14^ The consequential lipid metabolism dysregulation changes the phenotype of macrophages and impairs their function.^15^ There is growing recognition of the central role of rewiring cellular metabolic pathways in response to the extrinsic environment and intrinsic coordination.

Metabolic reprogramming similar to tumour cells has been observed in some immune cells residing in microenvironmental stimuli, including macrophages.^5^, ^10^ Early studies on metabolic reprogramming in cancer cells showed how metabolism regulates cell fate.^16^ Cancer cells autonomously alter the fluxes through various metabolic pathways to satisfy the demand for increased bioenergetics and biosynthesis.^16^, ^17^ Recent evidence suggests that metabolism plays a vital role in shaping the phenotype and function of macrophages.^18^ Under normal physiological and pathological conditions, macrophages are exposed to oxygen gradients. Macrophages adapt to hypoxia by shifting their metabolic pathway to glycolysis.^19^ After macrophage activation, their metabolic pathways, including gluconeogenesis, lipid metabolism and amino acid metabolism, are altered.^18^ As described earlier, macrophages are constantly exposed to lipid and metabolic stress by phagocytosis of lipids, accumulation of cellular cholesterol and transformation into foam cells until their eventual death. During this process, macrophages produce pro‐inflammatory factors.^15^, ^20^ Together with lipids, inflammatory factors and chemical properties in AS plaques, they create a complex microenvironment that perpetuates macrophage inflammation.

In this review, we provide a framework for understanding the intrinsic mechanisms and extrinsic pathways of macrophage metabolic reprogramming in the atherosclerotic unstable plaque microenvironment and whether these metabolic rewires, in turn affect the microenvironment. Furthermore, we discuss whether targeting these different pathways and microenvironmental changes in macrophages can alter the fate of vulnerable plaques.

## 2 OVERVIEW OF AS PLAQUE MICROENVIRONMENT AND MACROPHAGES

### 2.1 Distinct microenvironment in atherosclerotic plaques

**FIGURE 1:**
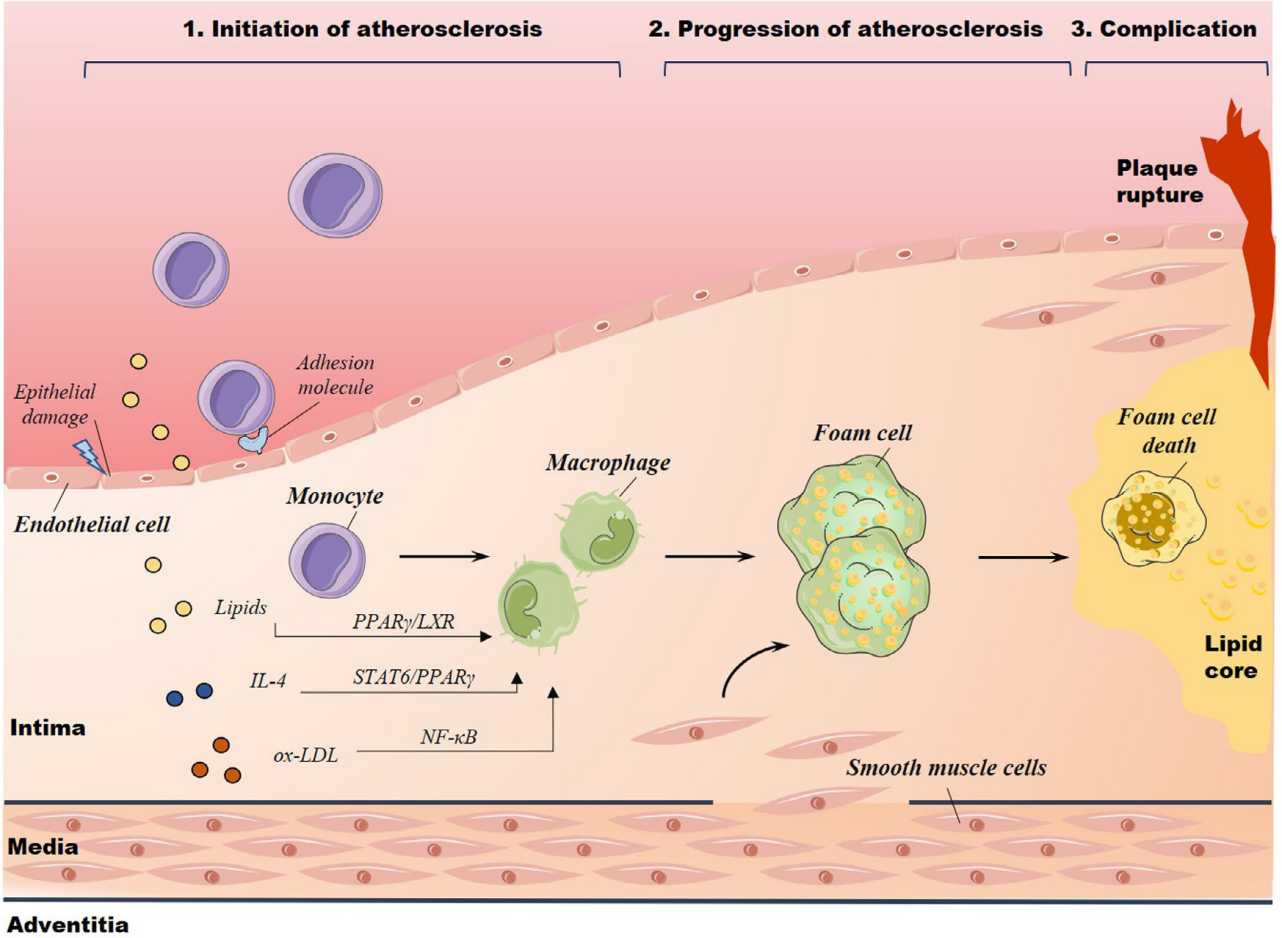
The pathogenesis of atherosclerosis (AS): initiation, progression and the complications of AS. In the initiation phase, activated endothelial cells express adhesion molecules that promote the rolling of circulating monocytes that adhere and migrate to the sub‐endothelium to become macrophages. Stimulation of triggers such as lipids, interleukin‐4 (IL‐4) and oxidised low‐density lipoprotein (ox‐LDL) leads to activation of transcription factors. During the evolution, macrophages and smooth muscle cells derive into foam cells. As the lesion progresses, foam cells can undergo cell death, including apoptosis, and form a lipid core. The advanced atherosclerotic plaques showed atheroma complications: plaque rupture and superficial erosion.

A complex immune and inflammatory response primarily mediates atherosclerotic plaque formation.^2^ The pathological processes of AS include three stages: initiation, progression and complications (Figure 1). With the progression of the disease, the plaque microenvironment is constantly changing.

#### 2.1.1 The initiation of AS

The ECs are disturbed when the intima is exposed to atherogenic risk factors, including oxidised and other modified low‐density lipoprotein (LDL) particles,^21^ pro‐inflammatory cytokines, local disturbances in the haemodynamic environment and cardiovascular‐related risk factors.^22^ Activated ECs express adhesion molecules (e.g., vascular cell adhesion molecule1 [VCAM‐1]) and chemokines that promote the rolling, adhesion of circulating monocytes to the endothelium and migration of bound monocytes into the arterial wall.^23^ Monocytes mature into macrophages once sub‐endothelium and acquire characteristics associated with anti‐ or pro‐inflammatory macrophage population.^24^

#### 2.1.2 The progression of atherosclerotic lesions

Atherosclerotic plaques develop through the continuous accumulation of lipids and foam cells.^25^ During the evolution of atherosclerotic plaques, smooth muscle cells, normally quiescent in the intima, migrate into the intima and become foam cells, similar to macrophages.^26^ Macrophages and smooth muscle cells can undergo programmed cell death such as apoptosis, forming a lipid core at the late stage of the plaque. Impaired clearance of dead cells, known as defective efferocytosis, may also lead to the formation of necrotic cores.^27^, ^28^

#### 2.1.3 The complications of AS: plaque rupture and superficial erosion

Atherosclerotic plaque rupture and surface erosion are the most common triggers of acute coronary artery thrombosis.^1^ ‘Vulnerable plaque’ refers to AS plaque with a large lipid core covered by a thin fibrous cap. Plaque rupture primarily involves a crack or fracture in the fibrous cap covering the lipid core of the atherosclerotic plaque.^4^, ^29^, ^30^, ^31^ Emerging evidence has addressed an alternative mechanism of plaque disruption (called surface erosion) increases. The pathological basis of this injury does not involve cracks or ruptures in the fibrous plaque cap but rather EC damage.^1^, ^29^, ^32^ The erosion mechanism involves polymorphonuclear leucocytes and neutrophil extracellular traps as local factors in thrombus propagation.^33^

#### 2.1.4 Plaque microenvironment

The concept of the atherosclerotic microenvironment is often used in the study of macrophage plasticity. The macrophages within the atherosclerotic plaque microenvironment are affected by various signals, such as oxidised lipids, differentiation factors and cytokines, that affect macrophage activation and polarisation, resulting in dynamic plasticity.^34^, ^35^ The plaque microenvironment is a highly complex physical and biochemical environment composed of various cells and molecules. These include multiple cells, such as ECs, monocytes/macrophages and VSMCs.^36^, ^37^ Several reports have shown that vulnerable plaque microenvironments exhibit low pH, excess reactive oxygen species (ROS) and lipid‐rich characteristics that distinguish them from stable plaques.^6^, ^38^ Microenvironments are crucial in shaping tissue‐resident macrophages’ unique identity and function.^8^, ^39^ Interestingly, when fully differentiated macrophages were transferred to the replacement tissues, the new environment was sufficient to reshape their expression. The macrophage chromatin landscape is specialised within tissues while retaining the ability to be reversed. ‘Understanding the composition of human atherosclerotic lesions has been challenging due to the complexity of human plaques and the lack of analytical methods’, says Giannarelli.^40^ However, advances in technological research, such as single‐cell sequencing, can advance our understanding of this disease.

### 2.2 Macrophages in AS: dynamic balance

Macrophages are involved in the initiation, progression and regression of AS. The focus is on how macrophage recruitment, polarisation and foam cell formation alter the fate of plaques.

#### 2.2.1 Mechanisms of macrophage accumulation

Recruiting monocytes to AS‐prone sites is an early step in AS.^41^ Transendothelial migration of monocytes is mediated by adhesion molecules secreted by ECs, macrophages and smooth muscle cells, such as intercellular adhesion molecule 1 (ICAM‐1), VCAM‐1 and selectins. Macrophages rely on scavenger receptor pathways and other mediators to uptake natural and modified LDL.^42^, ^43^, ^44^

#### 2.2.2 Macrophage polarisation and plasticity

Macrophages are remarkably plastic cells that exhibit specific phenotypes responding to different microenvironmental stimuli.^11^ In AS, macrophage heterogeneity is a now‐accepted concept. In addition to the classical M1–M2 classification, with the advancement of single‐cell sequencing and other technologies, it has been found that a large number of macrophages with different phenotypes are simultaneously present in atherosclerotic plaques.^45^ Different macrophage subclasses were identified in the plaques, including M1, M(Hb), Mhem, M2, M4 and Mox.^46^ Lipopolysaccharide (LPS), pro‐inflammatory cytokines, cholesterol crystals and ox‐LDL can stimulate M1 macrophages in atherosclerotic lesions.^47^ Interleukin (IL)‐4, IL‐13 and IL‐10 cytokines can activate M2 macrophages.^46^ Histological analysis of human plaques reveals that the number and presence of macrophages in plaques varies, with M1 macrophages mainly located in lipid‐rich areas away from M2 macrophages, and that these macrophages change their phenotypic expression over time, depending on the location and microenvironment, all of which regulate plaque progression and composition. The presence of oxidised phospholipids induces a phenotype called Mox macrophage, with increased expression of nuclear factor erythroid 2‐related factor 2‐dependent genes and ROS. In addition, M4 macrophages are induced by the platelet chemokine CXCL4 and expressing a combination of CD68, S100A8 and MMP7. They are mainly expressed in the outer and inner membranes and are associated with plaque instability.^25^, ^39^, ^48^ However, unlike in vitro experiments with a single inducing factor, in vivo microenvironment is complex and variable. Recently, single‐cell studies from human and murine AS have revealed the main macrophage populations of plaques.^49^, ^50^, ^51^ It is more conducive to identifying markers of different macrophage subsets and understanding the heterogeneity of macrophages in the microenvironment.

#### 2.2.3 Foam cell formation

The uptake of oxidised lipid particles by macrophages is considered the early event in plaque formation. The efficient esterification and cholesterol storage by macrophages as cholesteryl ester droplets can be viewed as a protective response.^52^ The mechanisms of foam cell formation have been intensively studied (see ref.^53^). Several transcription factors are essential in foam cell formation and maintenance.^8^ For example, ATF3 deletion induced foam cell formation and aggravated AS in mice.^54^ CCAAT enhancer‐binding proteins (C/EBPs) are involved in adipocyte differentiation and play a key role in macrophage polarisation.^55^, ^56^, ^57^ A study showed that C/EBPβ is enriched in open chromatin regions when macrophages were stimulated with ox‐LDL.^57^ These researches suggest a link between transcription factors and the development of AS.

## 3 MACROPHAGE METABOLIC REPROGRAMMING ENDOGENOUSLY DRIVEN

**FIGURE 2:**
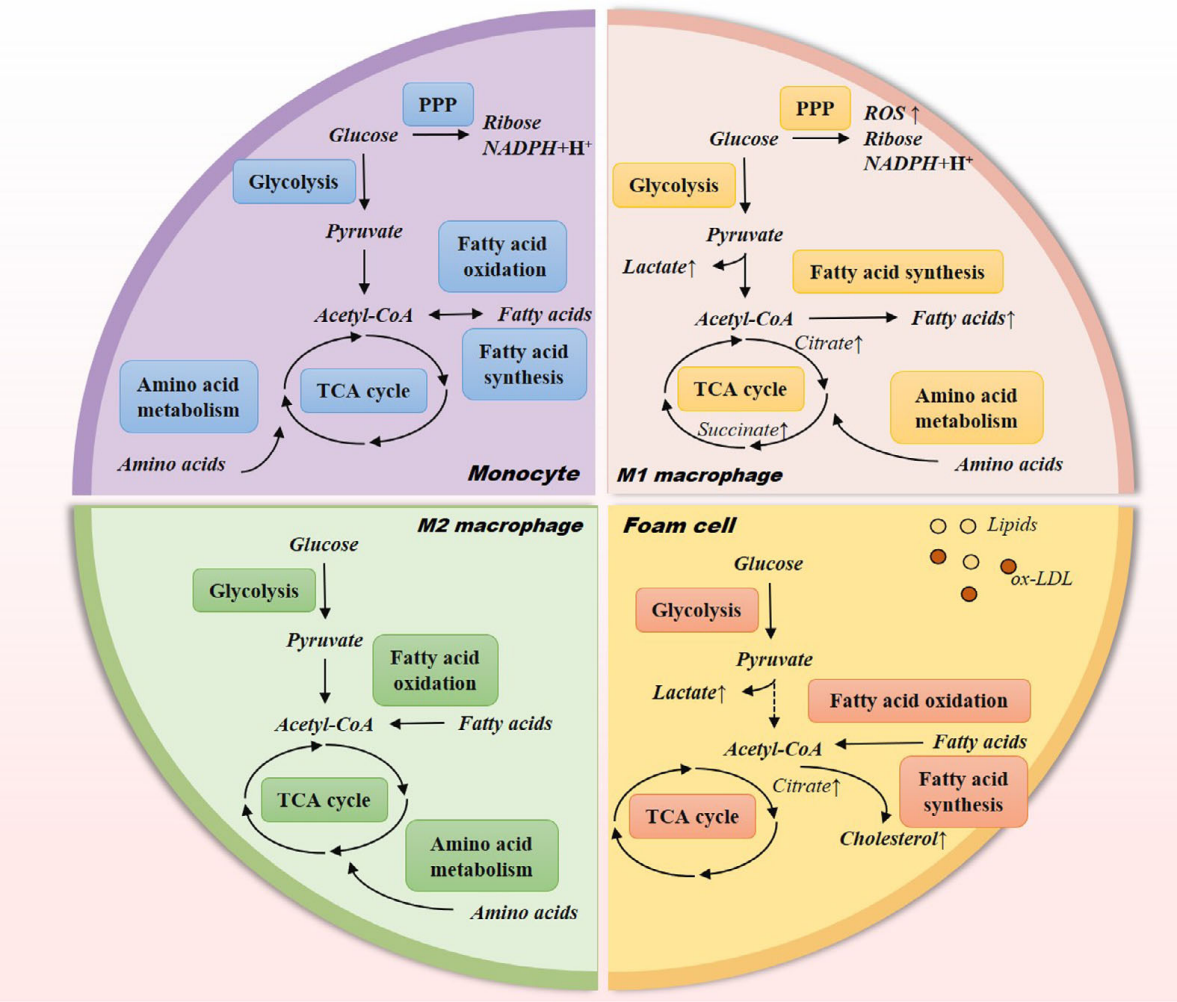
Six major metabolic pathways and metabolic reprogramming in monocytes, macrophages and foam cells. The critical metabolic pathways contained glycolysis, tricarboxylic acid (TCA) cycle, pentose–phosphate pathway (PPP), fatty acid oxidation (FAO), fatty acid synthesis and amino acid metabolism are intertwined pathways used to generate energy in monocytes. In M1 macrophages, the TCA cycle is impaired, and glucose metabolism tends towards glycolysis and the PPP—accumulation of lactate and reactive oxygen species (ROS), etc. Lipid metabolism is tilted towards fatty acid synthesis. Lactic acid accumulation also appeared in foam cells. M2 macrophages have an entire TCA cycle and can obtain energy through aerobic oxidation and FAO.

Intrinsic and extrinsic cellular signals interact to influence the rewiring of macrophage metabolic pathways. We focus on an overview of the metabolic reprogramming of the three major nutrients of macrophages, glucose, lipids and amino acids, and the changes in the effects of macrophages (Figure 2).

### 3.1 Reprogramming of glucose metabolism in macrophages

#### 3.1.1 Glucose metabolism

The primary biological function of glucose is to provide energy and carbon sources in the organism's metabolism. Among the catabolism of glucose, anaerobic oxidation is a relatively inefficient pathway for cellular adenosine triphosphate (ATP) production, including two stages: glycolysis and reduction of pyruvate to lactate. However, nicotinamide adenine dinucleotide (NADH)'s intermediate product produced during glycolysis is transferred to the biosynthetic growth pathway. Thus, glycolysis is dominant in the metabolism of rapidly proliferating cells. The tricarboxylic acid (TCA) cycle is the ultimate metabolic pathway for the three major nutrients and is the hub for the metabolic linkage of glucose, lipids and amino acids. The TCA cycle and oxidative phosphorylation (OXPHOS) are efficient ATP production modes that require mitochondrial biogenesis compared to glycolysis. Therefore, cells that require rapid ATP production will switch to glycolysis.^58^ The pentose–phosphate pathway (PPP), the primary source of NADPH and ribose phosphate, occurs in the cytoplasm. The PPP is more active in tumour tissues than in normal tissues, facilitating biosynthesis.^5^

#### 3.1.2 Reprogramming of glucose metabolism

Previous studies have investigated why tumour cells prefer glycolysis as a low‐productivity metabolism. This is reflected in two aspects: first, it provides a significant carbon source for synthesising proteins, lipids and nucleic acids; second, it shuts down aerobic oxidation pathways and reduces free radical production, thus evading apoptosis.^59^ Enhanced glycolysis allows immune cells to produce ATP rapidly and biosynthetic intermediates to perform specific effector functions. In the case of macrophages, this includes inflammatory cytokine production, and the transformation of different activation forms of macrophages.

The different activated forms of macrophages obtain energy through different glycolytic pathways. In M1 macrophages, glycolytic metabolism is enhanced, and the TCA cycle is impaired. The TCA cycle is disrupted at two sites after citrate and succinate.^60^, ^61^ The citrate accumulation causes nitric oxide (NO) production and inactivation of the electron transport chain.^62^, ^63^ Moreover, the second break of the TCA cycle contributes to NO production via an enhanced arginosuccinate shunt.^60^ In addition, accumulated citrate is exported from mitochondria via citrate transporter proteins involved in fatty acid production.^61^ Activated M1 macrophages upregulate succinate content, and the increase in succinate stabilises hypoxia‐inducible factor 1‐alpha (HIF‐1α) and further promotes glycolysis.^64^, ^65^ HIF‐1α is a transcription factor essential for the induction of several enzymes involved in glycolysis, and it may also be involved in the activation of nuclear factor‐kappa B (NF‐κB).^64^ High expression of glucose‐6‐phosphate dehydrogenase (a key enzyme of PPP) also activates the NF‐κB signalling pathway, causing intracellular oxidative stress and inducing M1 macrophage polarisation.^61^, ^66^ In contrast, M2 macrophages have an entire TCA cycle and can obtain the energy they need through aerobic oxidation. Studies have shown that inhibition of HIF‐1α converts macrophages from M1 phenotype to M2 phenotype.^19^ The PPP produces ribose, which is used for nucleotide synthesis for DNA and RNA and NADPH synthesis or to maintain the reduced state of glutathione (GSH) and promote antioxidant responses.

During glycolysis, methylglyoxal (MGO) is produced as a by‐product. MGO is a highly active dicarbonyl compound that can react with proteins quickly to form advanced glycation end products (AGEs). MGO has been found in atherosclerotic plaques. According to the immunohistochemistry of human atherosclerotic plaques, AGEs were mainly concentrated in macrophages around the necrotic core.^67^ Classically activated mouse macrophages produce MGO, which induces TLR4‐ and RAGE‐independent pro‐inflammatory responses.^68^ The details of MGO in macrophages metabolic reprogramming are reviewed in detail.^69^ However, the causal relationship between MGO, AGEs and macrophages in AS needs further study.

### 3.2 Reprogramming of lipid metabolism in macrophages

#### 3.2.1 Lipid metabolism

Fatty acid metabolism is an essential form of cellular lipid metabolism and one of the pathways of cellular energy sources. Fatty acids enter the mitochondria after activation and complete oxidation through repeated cycles of four‐step reactions of dehydrogenation, water addition, re‐dehydrogenation and thiolysis to produce acetyl coenzyme A, which is finally wholly oxidised through the TCA cycle and releases large amounts of energy. One portion of fatty acids can produce more than 100 molecules of ATP. The NADPH hydrogen supply performs fatty acid synthesis, and fatty acid is synthesised through multiple cycles of condensation, reduction, dehydration and re‐reduction reactions and re‐extension.^15^, ^66^

#### 3.2.2 Reprogramming of lipid metabolism

The activation of macrophages is closely related to the reprogramming of lipid metabolism. Lipidomic studies have elucidated the importance of lipid metabolism in the polarisation of macrophages to an inflammatory phenotype. Anti‐inflammatory M2 macrophages convert to fatty acid oxidation (FAO) and OXPHOS.^70^, ^71^ On the contrary, fatty acid synthesis positively regulates the generation and function of pro‐inflammatory M1 macrophages.^15^, ^72^ Hydrolysis products of LDL activate peroxisome proliferator‐activated receptor α (PPARα) and PPARγ, which inhibit the NF‐κB signalling pathway, suppress inflammatory signalling and contribute to macrophage polarisation towards the M2 type.^73^ In M1 macrophages, fatty acid synthase (FAS) is a key factor regulating fatty acid synthesis.^74^ Researchers have found that FAS deletion in macrophages prevents chronic inflammation in mice. In addition, the authors suggest that FAS is required for macrophage membrane remodelling and that FAS deficiency leads to alterations in plasma membrane composition and Rho GTPase trafficking, thereby blunting inflammatory signalling in macrophages.^75^ Beyond the initial stage, increased intake of lipids such as cholesterol increases the inflammatory marker molecule C‐reactive protein expression and affects macrophage activation.^76^

In recent years, this classical view has been challenged by recent findings. Recent evidence suggests that FAO is also essential for inflammasome activation in M1 macrophages, and glycolysis is now considered to contribute to FAO in M2 macrophages.^72^ The specific mechanisms by which differences in the metabolism of different fatty acids contribute to causing opposite activation patterns and different functions of macrophages need to be further investigated, but the different phenotypes of pro‐ and anti‐inflammatory phenotypes may influence different metabolic patterns due to their different dependence on energy requirements and conversion efficiency. Taken together, it is clear that lipid metabolic reprogramming plays an essential role in macrophage activation and maintenance.

Cholesterol processing by macrophages is dysregulated in AS. Due to the increased production of ox‐LDL, the scavenger receptor lectin‐like ox‐LDL receptor‐1 (LOX‐1) of macrophages is significantly upregulated in response to stimulation by various factors.^77^ For example, ox‐LDL, pro‐inflammatory cytokines and AGEs.^77^, ^78^ High expression of LOX‐1 leads to increased lipid uptake by macrophages. Furthermore, the reduced expression of ATP‐binding cassette transporter A1 (ABCA1) and ATP‐binding cassette transporter G1 (ABCG1) in AS further aggravated the accumulation of intracellular cholesterol and promoted foam cell formation.^79^

### 3.3 Reprogramming of amino acid metabolism in macrophages

#### 3.3.1 Amino acid metabolism

Amino acids undergo dynamic changes in catabolism, metabolic transformation and acquisition. Amino acids can be used as raw materials for proteins and are closely associated with crucial anabolic cell signalling pathways, especially the mechanistic target of rapamycin (mTOR) pathway, and nucleotide synthesis.^80^ They can be transformed into various physiologically important active substances or produce specific chemical groups through proper catabolism. It can also be oxidised by the TCA cycle to produce energy, carbon dioxide and water.^81^

#### 3.3.2 Reprogramming of amino acid metabolism

Amino acid anabolism, catabolism and material transformation and reprogramming play essential roles in macrophage activation and function. Through proper metabolic conversion, amino acids are an essential source of raw materials for bioactive substances such as intracellular nitrogen‐containing compounds, such as NO, porphyrin compounds and bases. Arginine metabolism has long been considered an essential metabolic regulator for macrophage polarisation and inflammatory responses.^82^ In macrophages, arginine metabolic pathways mediated by inducible nitric oxide synthase (iNOS) and arginase play crucial roles in M(IFN‐γ) and M(IL‐4) processes, respectively. Arginine catalysed by NOS generates NO, and M1 macrophages highly express iNOS that catalyse arginine synthesis of NO, which is required for inflammatory macrophage function.^83^ The arginase pathway inhibits NO production by reducing arginine in comparison to the inflammatory response. Arginase, highly expressed in M2 macrophages, catalyses the hydrolysis of arginine and regulates macrophage proliferation and collagen synthesis.^83^, ^84^

Among amino acid metabolism, glutamine metabolism is vital in regulating immune cell function. Glutamine metabolism is also crucial for NO production through the synthesis of arginine input, demonstrating glutamine's role in macrophage cytotoxicity and antimicrobial function.^85^ Another feature distinguishing M2 macrophages from M1 macrophages is the increased glutamine metabolism. Researchers found that α‐ketoglutarate produced by glutamine catabolism promotes epigenetic activation of M2‐type genes in macrophages, whereas glutamine is not required for LPS‐stimulated M1 macrophage development.^60^ Interestingly, a recent study has shown that impaired macrophage glutamine breakdown exacerbates AS. High‐throughput transcriptional and metabolic profiling revealed that the phagocytic capacity of macrophages was dependent on the atypical transaminase pathway, but not on the traditional glutamate dehydrogenase (GLUD1) to support α‐ketoglutarate‐dependent immune metabolism.^86^

In addition, recent studies have found that serine and citrulline are associated with macrophage activation. Shan et al.^87^ showed that inhibition of serine metabolism, either by inhibiting the activity of phosphoglycerate dehydrogenase, a key enzyme in the serine biosynthesis pathway, or by limiting exogenous serine and glycine, significantly enhanced the polarisation of M(IFN‐γ). However, it inhibited the polarisation of M(IL‐4). Investigators have revealed a central role for the urea cycle metabolic enzyme ASS1 in controlling inflammatory macrophage activation and antimicrobial defense by depleting cellular citrulline.^88^

## 4 MICROENVIRONMENT SHAPE PLAQUE MACROPHAGE METABOLISM AND FUNCTION

**FIGURE 3:**
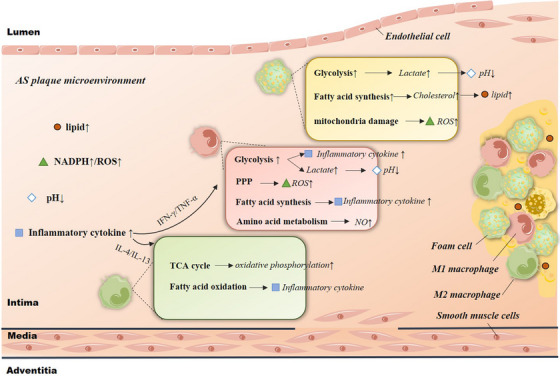
Macrophages metabolic co‐dependencies in the atherosclerosis (AS) plaque microenvironment. The vulnerable plaque microenvironments exhibit low pH, excess reactive oxygen species (ROS), lipid‐rich and inflammatory factors characteristics. Microenvironment changes induce macrophage activation and foam cell transformation. Simultaneously, macrophage heterogeneity affected the changes of the above factors.

In atherosclerotic plaques, the microenvironmental cues trigger the activation or polarisation of macrophages. These forces vary with the evolution and progression of the plaque. Macrophages are exposed to various biological factors and changing biochemical conditions, including cytokines, lipids, cholesterol crystals, oxidative stress components such as ROS, and pH reduction. However, few reviews have described the effects of the above AS microenvironmental factors on the metabolic reprogramming of macrophages. We summarise the effect of biological and biochemical environmental changes in the microenvironment on macrophage activation, function and metabolic reprogramming (Figure 3).

### 4.1 Effect of cytokines on macrophage metabolic reprogramming

In AS, cytokines can be broadly classified as pro‐ or anti‐atherosclerotic.^89^ Pro‐inflammatory cytokines, such as tumour necrosis factor‐alpha (TNF‐α) and IL‐1, are mainly mediated by the NF‐κB pathway, affecting almost all cells involved in AS formation. Anti‐atherosclerotic cytokines differ from the above and protectively prevent atherosclerotic plaque formation. These include transforming growth factor‐β, IL‐10, etc. Among them, IL‐10 can exert anti‐atherosclerotic activity by downregulating TNF‐α production and ICAM‐1 expression on ECs.^34^, ^90^

Macrophages are exposed to the AS microenvironment, and the local cytokine environment can affect macrophage polarisation. Specifically, activating macrophages to the M1 state by inflammatory stimuli such as interferon‐gamma (IFN‐γ) is associated with enhanced glycolytic metabolic pathways.^91^ The enhanced glycolytic metabolic pathway was associated with 6‐phosphofructo‐2‐kinase (PFK2). Phosphofructokinase‐1 (PFK) is most important for regulating glycolysis flow. Furthermore, PFK2 is the catalyst of fructose‐2,6‐biphosphate, the most potent allosteric activator of PFK. IFN‐γ stimulation shifts the expression of liver PFK2 to the more active and ubiquitous PFK2 isoform (uPFK2), which maintains a higher fructose‐2,6‐biphosphate concentration.^91^, ^92^ The increased glycolytic rate allows M1 macrophages to rapidly produce sufficient energy and biosynthetic intermediates to rapidly perform to cope with the hypoxic microenvironment.^60^

In contrast to IFN‐γ stimulation, macrophages mainly rely on the TCA cycle pathway and OXPHOS for glucose metabolism and energy supply after IL‐4 stimulation.^93^ Metabolomic analysis of M2 macrophages showed that the TCA cycle contributes to the production of uridine diphospho‐N‐acetylglucosamine (UDP‐GlcNAc) intermediates, which are associated with the clearance function of M2.^60^, ^94^ Another study reinforced the link between mitochondrial OXPHOS and M2 polarisation, where iNOS inhibited OXPHOSP in M1 macrophages, promoting their metabolic and phenotypic reprogramming to the M2 phenotype.^95^ Further molecular studies of metabolic reprogramming in M2 macrophages suggest that IL‐4 triggers PPARγ coactivator 1β (PGC‐1β) in these cells, which is responsible for converting mitochondrial respiration and FAO.^96^

Interestingly, further studies have found that foam cell formation may locally attenuate the macrophage‐dependent inflammatory component in atherogenesis.^97^ Human monocytes were cultured in vitro and transformed into foam cells by treatment with macrophage colony‐stimulating factor (M‐CSF) and acetylated LDL. When stimulated by LPS and IFN‐γ, compared with M1 macrophages, the expression of pro‐inflammatory genes was decreased, the secretion of TNF‐α was reduced and the NF‐κB showed a downwards trend. When subjected to alternate M2 polarisation, macrophages and foam cells respond by upregulating typical anti‐inflammatory genes, which are equal to both cell types.

### 4.2 Effect of microenvironmental lipid changes on macrophage metabolic reprogramming

One of the characteristics of AS is the increased permeability of the vascular EC barrier to ox‐LDL. Macrophages reside in the plaque microenvironment and attempt to remove inflammatory lipids and transform them into macrophage‐derived foam cells. The balance and imbalance of macrophage cholesterol in AS have been reviewed.^15^, ^98^ Briefly, LDL receptor‐mediated cholesterol uptake. ABCA1/ABCG1 mediated reverse cholesterol transport. Dysregulation of the macrophage surface receptor CD36 leads to increased uptake of ox‐LDL.^99^, ^100^ ox‐LDL strongly affects the polarisation of macrophages and the secretion of inflammatory factors. It has been found that LDL could induce the polarisation of macrophages to pro‐inflammatory phenotype.^101^ Moreover, activation of the NF‐κB pathway promotes pro‐inflammatory factor secretion and macrophage inflammatory response.^102^, ^103^ Oxidised phospholipids were found to trigger the Mox phenotype of macrophages by activating the Nrf2 transcription factor in a mouse model.^104^

Macrophage lipidomic analysis further elucidated the differential expression and metabolic changes of various lipid mediators and their relationship with macrophage function.^105^ Using lipidomic and transcriptomic approaches, Spann et al.^14^ demonstrated that regulated desmosterol accumulation underlies many homeostatic responses in macrophage foam cells, including activation of LXR target genes, selective reprogramming of fatty acid metabolism, and repression of inflammatory response genes. These findings suggest that macrophage activation in atherosclerotic lesions is caused by exogenous microenvironment signalling inhibition that inhibits desmosterol homeostasis and anti‐inflammatory functions.

### 4.3 Effect of oxidative stress on macrophage metabolic reprogramming

All redox reactions occurring in living organisms are collectively referred to as biological oxidation. Nutrients undergo dehydrogenation reactions via other metabolic pathways, such as the TCA cycle, producing hydrogen atoms as reduced equivalents NADH + H^+^ or FADH_2_. The respiratory chain is also the body's most important source of ROS, and excessive ROS accumulation can harm the organism. ROS production and ongoing inflammation may lead to eventual thinning of the fibrous cap on the plaque.^106^ There are several crucial ROS‐generating systems in the microenvironment, and these pro‐oxidant systems crosstalk with each other.^107^ These include NADPH oxidase, xanthine oxidase, the mitochondrial enzyme respiratory chain, and dysfunctional, uncoupled NOS.^107^ Elevated ROS and low oxygen concentration drive macrophage metabolic reprogramming, enhancing cellular dependence on glycolysis.

A high concentration of ROS microenvironment is formed within the plaques with upregulation of multiple inflammatory markers, such as iNOS.^108^ Cellular glucose uptake is increased, and glycolytic metabolic pathways are enhanced. iNOS catalyses NO synthesis from arginine and participates in macrophage pro‐inflammatory responses.^109^

## 5 MACROPHAGE REPROGRAMMING COUNTERACTS THE MICROENVIRONMENT

Macrophages are dynamically changing, and as they undergo metabolic reprogramming in the microenvironment to make plastic and heterogeneous changes, they also shape the plaque microenvironment in reverse. In the following, we will describe the counter‐actions of macrophage reprogramming on the microenvironment in terms of macrophage cholesterol export, cytokine secretion, biological enzyme changes, pH and other chemical environment regulations (Figure 3).

### 5.1 Macrophage cholesterol export

Macrophage uptake of lipoproteins under steady‐state conditions and lipoproteins are transported to endosomes/lysosomes for hydrolysis and metabolism. Furthermore, cytoplasmic cholesterol can be transported to the endoplasmic reticulum by re‐esterification and stored in lipid droplets or excluded from the cell across the cell membrane. Macrophages in advanced plaques showed a considerable accumulation of free cholesterol, thus indicating macrophage failure and a malfunction in the processes that maintain cholesterol homeostasis in macrophages.^5^ It was found that mouse macrophages lacked PGC‐1α, an essential booster protein for mitochondrial function, and exhibited impaired cholesterol efflux. Macrophages export high‐density lipoproteins via the ABCA1 and ABCG1 pathways to remove excess cholesterol.^110^ In vitro studies showed that the function of ABCA1, a cholesterol efflux transporter, was significantly reduced in macrophages in association with HIF‐1α under hypoxia, further exacerbating plaque macrophages cholesterol accumulation.^111^ Therefore, lipid overload of foam cells leads to apoptosis and necrosis, forming free cholesterol crystals and hypoxia in the microenvironment, further aggravating the imbalance of cholesterol metabolism.

### 5.2 Cytokine secretion

In the progression of AS, macrophages play an essential role as central elements in the secretion of inflammatory cytokines to induce an inflammatory microenvironment. Increased pro‐inflammatory factors increase the risk of rupture of vulnerable plaques.^39^ As mentioned, macrophages in atherosclerotic plaques are affected by the microenvironment, which alters their functional phenotype and releases different cytokines.

Inflammasomes are intracellular complexes involved in innate immunity. The NLRP3 inflammasome drives the activation of caspase‐1 to produce IL‐1β and IL‐18.^112^ NLRP3 is considered to be the link between lipid metabolism and inflammation. Our group verified that ox‐LDL could activate the NLRP3 inflammasome in macrophages.^113^ In addition, NLRP3 can also be triggered by crystalline cholesterol.^112^ Components of glycolysis (e.g., hexokinase) are involved in the regulation of NLRP3, which affects the release of IL‐1β and IL‐18.^114^ In M1 macrophages and foam cells, the TCA cycle is impaired, and accumulated succinate further stabilises HIF‐1α expression and induces pro‐inflammatory cytokine IL‐1β expression.^64^, ^115^, ^116^ Serine is a substrate for synthesising nucleotides, NADPH and GSH. Previous study reveals that serine metabolism is necessary for GSH synthesis to support IL‐1β cytokine production.^117^

### 5.3 Microenvironmental pH and other chemical environment regulation

The microenvironment of atherosclerotic plaques is acidic. It is generally believed to be related to anaerobic metabolism and lactate formation. Further investigation revealed extensive macrophage and foam cell infiltration in vulnerable plaques prone to rupture. Macrophages are very metabolically active, especially preferring glycolysis to synthesise ATP under hypoxic conditions. Thus, local production and secretion of lactate and H^+^ are enhanced.^5^ In addition to this, a negative correlation between temperature and pH was found.^38^ It may be related to the production of heat during macrophage metabolism. Finally, decreased ABCA1 expression at acidic pH may affect cholesterol clearance in atherosclerotic lesions. In conclusion, acidic extracellular pH amplifies the pro‐atherogenic and pro‐inflammatory processes associated with AS.

## 6 MACROPHAGE METABOLIC REPROGRAMMING AND ATHEROSCLEROTIC PLAQUES MICROENVIRONMENT: AS DIAGNOSTIC AND THERAPEUTIC STRATEGY

Assessing atherosclerotic plaque susceptibility and early intervention is essential to reducing cardiovascular disease mortality. We review the diagnosis and treatment of plaque microenvironment and macrophages, respectively.

### 6.1 Diagnostic and therapeutic strategies for plaque microenvironment

In response to lipid oxidation reactions, weakly acidic microenvironmental changes and macrophage changes in multiple phenotypes, various nanoagents and others are involved in precise imaging or treating vulnerable atherosclerotic plaques.

#### 6.1.1 Diagnostic strategies

**FIGURE 4:**
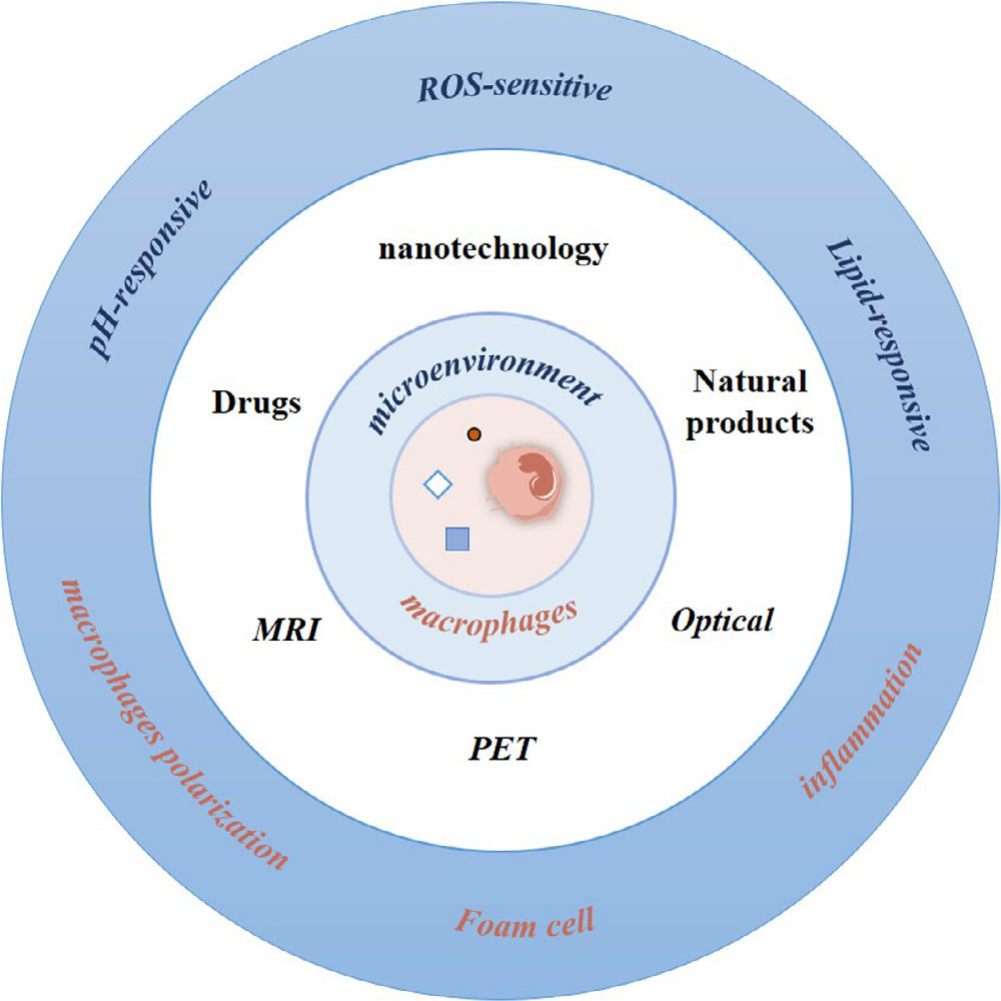
Diagnostic and therapeutic maps of the atherosclerotic plaque microenvironment and macrophages. It sequentially shows three therapeutic modalities (drugs, natural products and nanotechnology) and three imaging modalities (magnetic resonance imaging [MRI], positron emission tomography [PET] and optical) for microenvironment and macrophages. The outermost circle shows the different treatment strategies.

Atherosclerotic plaque formation is a chronic inflammatory process. Increased release of lactic acid, including macrophages, increases the number of factors contributing to a weakly acidic extracellular microenvironment. In areas of vulnerable plaque lesions the pH has been reported to be acidic (pH 5.5). Furthermore, the pH of lysosomes in macrophages is as low as 4.5–5.5.^6^ It was found that by exploiting the microenvironment and the acidic conditions in macrophage lysosomes, an iron oxide nanoprobe was developed for magnetic resonance imaging (MRI), thus facilitating the diagnosis of vulnerable plaques.^118^ AS plaque microenvironment is rich in lipids, part of which is ox‐LDL‐induced EC damage, adhesion molecule expression, etc., causing ox‐LDL deposition in the microenvironment; the other part is foam cell loss of compensation, impaired cholesterol metabolism, foam cell apoptosis, necrosis, which gradually forms lipid necrotic core. The problems of drug leakage, accumulation efficiency and specific recognition of nanocapsules were addressed. Studies use the erythrocyte membrane to obtain surface biomimetic nanoparticles that, in the case of ROS reactivity, are interrupted in inflamed atherosclerotic tissue and can be used for accurate anti‐inflammatory and lipid‐specific fluorescence imaging^119^ (Figure 4).

#### 6.1.2 Therapeutic strategies

Based on this low pH property of the plaque microenvironment, a pH‐sensitive functionalised red blood cell nanoplatform has been constructed with docetaxel as a model drug to treat targeted AS.^120^ The study found that an acid‐labile β‐cyclodextrin material (acetalated β‐cyclodextrin, Ac‐bCD) and a ROS‐sensitive β‐cyclodextrin material (Ox‐bCD), which were chemically modified with β‐cyclodextrin, were loaded with the anti‐AS drug rapamycin, which was verified to delay the progression of AS in ApoE^‐/‐^ mice. Moreover, it significantly enhanced the stability of atherosclerotic lesions^121^ (Table 1).

**TABLE 1:** Strategies for the therapy of plaque microenvironment and macrophages.

Targeting strategies	Drug	Target protein	Metabolic pathway	Reference
pH‐responsive	Ac‐bCD	Rapamycin	Suppress foam cell formation	^121^
	Erythrocyte platform	Docetaxel	Macrophage proliferation ↓	^120^
ROS‐sensitive	Ox‐bCD	Rapamycin	Suppress foam cell formation	^121^
Lipid‐responsive/macrophage	Statins	PPARγ/SR‐A/ABCA1	Lipid metabolism/atherosclerosis ↓	^125^
Macrophage	Metformin	AMPK/ATF1/HO‐1	Mhem phenotype ↑	^131^
	Pioglitazone	PPARγ	M2 phenotype ↑	^132^
	Sitagliptin	SDF‐1/CXCR4	M2 phenotype ↑	^133^
	Curcumin	CD36/SR‐A	Lipid metabolism/atherosclerosis ↓	^126^, ^134^, ^135^
	Polyphenols	ABCA1	Lipid metabolism/atherosclerosis ↓	^136^
	Glucocorticoid	PPARγ	the clearance of apoptotic cells	^104^
	miR‐33	ABCA1	Cholesterol efflux ↓	^129^
	CD11b‐NanoLIF	CD11b	M1 cell proliferation ↓	^127^
	mDNP‐LXR‐L	ABCA1/ABCG1	Lipid metabolism/atherosclerosis ↓	^128^

Abbreviations: ABCA1, ATP‐binding cassette transporter A1; ABCG1, ATP‐binding cassette transporter G1; Ac‐bCD, acetalated β‐cyclodextrin; mDNP, mannose‐functionalised dendritic nanoparticles; Ox‐bCD, ROS‐sensitive β‐cyclodextrin; ROS, reactive oxygen species; AMPK, AMP‐activated protein kinase; ATF1, activating transcription factor 1; heme oxygenase (HO)‐1.

### 6.2 Diagnostic and therapeutic strategies for macrophages

Macrophages play a crucial role in AS plaque progression and rupture of vulnerable plaques. The high plasticity of macrophages makes them an ideal target for diagnosing and treating AS.

#### 6.2.1 Diagnostic strategies

According to the different polarisation phenotypes of macrophages, various molecular markers and foam cell turnover, there are a variety of diagnostic markers and molecular probes for plaques. In vulnerable plaques, M1 macrophages are mostly present, and targeting macrophage heterogeneity suggests that predicting plaque vulnerability is a promising diagnostic method. 18F‐fluorodeoxyglucose visualises metabolic activity, binds to a macrophage‐like marker (CD68) and can be used for carotid detection.^122^ It has been reported that the macrophage receptor with collagen structure (MARCO) is significantly upregulated on the surface of M1 macrophages and ruptured plaques. One study found that the MARCO‐targeted upconversion luminescence probe can be used for optical/MRI dual‐modality imaging of M1 macrophages.^123^ Further studies have innovated using a novel P2X7‐targeting positron emission tomography probe (18F‐FTTM) to identify vulnerable atherosclerotic plaques, which provides a powerful non‐invasive method for diagnosing AS and the screening of new drugs for precision treatment.^124^

#### 6.2.2 Targeted macrophage therapy

With the advancement of drugs targeting metabolic reprogramming in oncology research, potential therapeutic targets targeting metabolic reprogramming in the development of AS have attracted interest. Targeted macrophage therapy mainly includes the following three aspects: affecting the macrophage polarisation, lipid metabolism reprogramming and inflammatory response of macrophages. Among them, lipid metabolism reprogramming of foam cells plays an anti‐AS strategy targeting lipid uptake, cholesterol esterification and cholesterol efflorescent pathways (Figure 4). In addition to natural products and drugs, nanotechnology also offers new perspectives. Hypoglycaemic drugs metformin, pioglitazone and sitagliptin could induce macrophages to change to M2 phenotype. It affects the phenotype of macrophages and plays an anti‐AS role. Natural pharmaceutical compounds, such as curcumin and polyphenols, could also induce macrophage polarisation (Table 1). Several other therapeutic strategies have been investigated for preventing foam cell formation using statins.^125^ Wang et al.^126^ elaborated on the therapeutic strategy of natural products targeting foam cells in the AS. However, natural products also have the disadvantages of low bioavailability, low potency and lack of specificity. Now, new drug‐delivery systems such as nanoparticles, stents, oligopeptide complexes, liposomes and monoclonal antibodies can selectively modify macrophages. To achieve macrophage‐targeted drug delivery in atherosclerotic plaques in vivo by targeting macrophage surface markers (CD11b, CD163, CD206).^104^ For example, it was found that encapsulation of leukaemia inhibitory factor by nanoparticles, coupled to CD11b antibody, delivered anti‐inflammatory cytokines to macrophages and promoted anti‐inflammatory transition.^127^ Mannose‐functionalised dendritic nanoparticles (mDNP) coated with LXR ligands (LXR‐L) were synthesised by polyamidoamine dendrimer molecules. mDNP‐LXR‐L could be effectively taken up by macrophages, increase the expression of LXR target genes (ABCA1/ABCG1) and promote cholesterol efflux.^128^

As an independent layer of metabolic regulation, non‐coding RNAs provide a potential therapeutic target for targeting macrophages. Among them, in lipid metabolism, a variety of microRNAs are involved in regulating cholesterol efflux by targeting ABCA1/ABCG1.^126^ For example, miR‐33 inhibits ABCA1 expression, thereby reducing cholesterol efflux.^129^ Anti‐miR33 nanotherapy can modulate the polarisation of macrophages to an M2 phenotype.^130^ These findings suggest that targeting macrophage microRNAs may be a promising therapeutic agent for AS.

### 6.3 Challenges and prospects

Presently, the treatment of AS includes the regulation of blood lipids and anti‐platelet aggregation. Statins are the cornerstone of the drug treatment of dyslipidemia, which can stabilise plaque and play an essential role in preventing and controlling atherosclerotic cardiovascular disease at home and abroad. However, the significant cardiovascular risk remains. To reduce the adverse reactions caused by drugs and improve the efficiency of treatment, it is of great significance to explore new forms of drug administration to intervene in AS. Over the past two decades, the rapid development of nanotechnology and bioengineering has strongly promoted the research on novel nanotherapeutic strategies for treating cardiovascular diseases. However, clinically approved nanotherapeutic agents are still scarce.^137^ In this review, we review some of the nanoparticles targeting the plaque microenvironment and macrophages. Newer nanoparticles offer better targeting beyond traditional structural imaging to achieve preclinical identification of non‐narrow and prone‐to‐rupture plaques. However, because macrophages are important immune system molecules, higher requirements are put forward for precision. With the in‐depth development of single‐cell sequencing, spatial omics and metabolomics technology, it is helpful to understand the vulnerable plaque microenvironment, cell‐to‐cell crosstalk between macrophages and other immune cells and lay the foundation for targeted therapy. The effectiveness of nanoparticle drug delivery systems has mostly been demonstrated in experiments, but it is not necessarily successful in humans. Human plaques are more complex because they develop over the years or even decades. Clinical translation remains a significant challenge due to problems in biocompatibility, low target efficiency and drug retention in the body. Unlike the industrial nano‐targeting platform, the exosome‐drug‐loaded targeting platform has better biocompatibility and low immunogenicity. However, the main clinical challenges are the imperfection and repeatability of the exosome extraction process.

## 7 CONCLUSIONS

In conclusion, this review focused on the mechanisms of intrinsic changes and extrinsic alterations in macrophage metabolic reprogramming in AS unstable plaques and their interaction with the AS unstable plaque microenvironment, focusing more on diagnostic and therapeutic strategies targeting the microenvironment and macrophage metabolism. The future of cellular metabolic therapy will be realised by fully understanding the complex heterotypic interactions in the AS microenvironment and redundant mechanisms controlling cellular metabolic co‐dependence.

## CONFLICT OF INTEREST STATEMENT

The authors declare no conflicts of interest.
